# *CBS* mutations are good predictors for B6‐responsiveness: A study based on the analysis of 35 Brazilian Classical Homocystinuria patients

**DOI:** 10.1002/mgg3.342

**Published:** 2018-01-20

**Authors:** Soraia Poloni, Fernanda Sperb‐Ludwig, Taciane Borsatto, Giovana Weber Hoss, Maria Juliana R. Doriqui, Emília K. Embiruçu, Ney Boa‐Sorte, Charles Marques, Chong A. Kim, Carolina Fischinger Moura de Souza, Helio Rocha, Marcia Ribeiro, Carlos E. Steiner, Carolina A. Moreno, Pricila Bernardi, Eugenia Valadares, Osvaldo Artigalas, Gerson Carvalho, Hector Y. C. Wanderley, Johanna Kugele, Melanie Walter, Lorena Gallego‐Villar, Henk J. Blom, Ida Vanessa D. Schwartz

**Affiliations:** ^1^ Post‐Graduation Program in Genetics and Molecular Biology Universidade Federal do Rio Grande do Sul Porto Alegre Brazil; ^2^ Laboratory of Basic Research and Advanced Investigations in Neurosciences (BRAIN) Hospital de Clínicas de Porto Alegre Porto Alegre Brazil; ^3^ Complexo Hospitalar Materno‐Infantil do Maranhão São Luis Brazil; ^4^ Complexo Hospitalar Professor Edgard Santos Universidade do Estado da Bahia Salvador Brazil; ^5^ Universidade do Estado da Bahia Salvador Brazil; ^6^ Hospital das Clínicas de Ribeirão Preto Ribeirão Preto Brazil; ^7^ Universidade de São Paulo São Paulo Brazil; ^8^ Medical Genetics Service Hospital de Clínicas de Porto Alegre Porto Alegre Brazil; ^9^ Universidade Federal do Rio de Janeiro Rio de Janeiro Brazil; ^10^ Universidade Estadual de Campinas Campinas Brazil; ^11^ Universidade Federal de Santa Catarina Florianópolis Brazil; ^12^ Universidade Federal de Minas Gerais Belo Horizonte Brazil; ^13^ Children′s Hospital Grupo Hospitalar Conceição Porto Alegre Brazil; ^14^ Genetics Unit Hospital Materno‐Infantil Presidente Vargas Porto Alegre Brazil; ^15^ Hospital de Apoio de Brasília Brasília Brazil; ^16^ Escola Superior de Ciências da Santa Casa de Misericórdia de Vitória Vitória Brazil; ^17^ Laboratory for Clinical Biochemistry and Metabolism University Medical Center Freiburg Germany

**Keywords:** classical homocystinuria, CβS deficiency, CβS expression, *CBS* mutations, homocysteine

## Abstract

**Background:**

Classical homocystinuria (HCU) is a monogenic disease caused by the deficient activity of cystathionine β‐synthase (CβS). The objective of this study was to identify the *CBS* mutations in Brazilian patients with HCU.

**Methods:**

gDNA samples were obtained for 35 patients (30 families) with biochemically confirmed diagnosis of HCU. All exons and exon‐intron boundaries of *CBS* gene were sequenced. Gene expression analysis by qRT‐PCR was performed in six patients. Novel missense point mutations were expressed in *E. coli* by site‐directed mutagenesis.

**Results:**

Parental consanguinity was reported in 16 families, and pyridoxine responsiveness in five (15%) patients. Among individuals from the same family, all presented the same phenotype. Both pathogenic mutations were identified in 29/30 patients. Twenty‐one different mutations were detected in nine exons and three introns; being six common mutations. Most prevalent were p.Ile278Thr (18.2%), p.Trp323Ter (11.3%), p.Thr191Met (11.3%), and c.828+1G>A (11.3%). Eight novel mutations were found [c.2T>C, c.209+1delG, c.284T>C, c.329A>T, c.444delG, c.864_868delGAG c.989_991delAGG, and c.1223+5G>T]. Enzyme activity in *E. coli‐*expressed mutations was 1.5% for c.329A>T and 17.5% for c.284T>C. qRT‐PCR analysis revealed reduced gene expression in all evaluated genotypes: [c.209+1delG; c.572C>T]; [c.2T>C; c.828+1G>A]; [c.828+1G>A; c.1126G>A]; [c.833T>C; c.989_991delAGG]; [c.1058C>T; c.146C>T]; and [c.444delG; c.444delG]. The expected phenotype according to the genotype (pyridoxine responsiveness) matched in all cases.

**Conclusions:**

Most patients studied were pyridoxine nonresponsive and presented early manifestations, suggesting severe phenotypes. Many private mutations were observed, but the four most prevalent mutations together accounted for over 50% of mutated alleles. A good genotype–phenotype relationship was observed within families and for the four most common mutations.

## 1 INTRODUCTION

Classical homocystinuria (HCU; OMIM 236200) is an inborn error of metabolism caused by deficient activity of cystathionine β‐synthase (CβS; EC 4.2.1.22). This enzyme catalyzes the first step of the transsulfuration pathway, whereby homocysteine is condensed with serine to form cystathionine. CβS deficiency leads to significantly elevated plasma levels of homocysteine and methionine and low levels of cysteine. HCU is inherited in an autosomal recessive pattern, and its worldwide prevalence is estimated between 1:100,000 and 1:344,000 (Moorthie, Cameron, Sagoo, Bonham, & Burton, 2014; Mudd, Levy, & Skovby, 2001; Skovby, Gaustadnes, & Mudd, 2010).

From a clinical standpoint, the classical signs of the disease are lens dislocation, thromboembolism, intellectual disability, psychiatric disorders, osteoporosis, and marfanoid features (Mudd et al., 1985, 2001). The treatment strategies include supplementation of pyridoxine (CβS cofactor), folic/folinic acid, betaine, and a methionine‐restricted diet (Morris et al., 2016; Schiff & Blom, 2012). Usually, patients who respond to pyridoxine supplementation exhibit a milder phenotype and have a better prognosis (Mudd et al., 1985; Skovby et al., 2010).

The *CBS* gene is located on chromosome 21q22.3. It spans 23 exons, with exons 1–16 comprising the coding region, which encodes a 551‐amino acid polypeptide. The 5′‐UTR region of the gene is formed by one of five alternative exons (−1a to −1e), in addition to exon 0. The 3′‐UTR region is encoded by exons 16 and 17 (Bao, Vlcek, Paces, & Kraus, 1998; Kraus et al., 1998). Over 160 different mutations in *CBS* have been reported, most of them being private. However, taken together, the four most prevalent mutations (p.Ile278Thr, p.Thr191Met, p.Gly307Ser, and p.Arg336Cys) account for more than half of all HCU alleles worldwide (Kraus, 2017). While the first of these mutations is panethnic, the other three follow rather well‐demarcated geographic and ethnic distributions (Cozar et al., 2011; El‐Said et al., 2006; Gallagher et al., 1995; Porto et al., 2005; Shih et al., 1995; Urreizti et al., 2006). The molecular bases of HCU in Brazil are poorly characterized. In the only study published (Porto et al., 2005), *CBS* analysis performed by RFLP and SSCP is reported for 14 patients (11 unrelated) followed in a single medical center located in southeast Brazil. The common mutations p.Ile278Thr and p.Thr191Met were detected at a frequency of 13.6% each.

A consistent genotype–phenotype correlation is described for some frequent mutations. For instance, p.Ile278Thr mutation is usually associated with milder phenotypes and pyridoxine responsiveness (Kraus, 2017; Shih et al., 1995). It is also reported that homozygotes for this mutation may have a higher risk of developing thromboembolism instead of other HCU symptoms (Magner et al., 2011; Skovby et al., 2010). Patients carrying the Latin/Iberian p.Thr191Met mutation are usually nonresponsive to pyridoxine, but a great variability of severity and clinical symptoms can be observed (Cozar et al., 2011; Urreizti et al., 2006).

The present study sought to establish a broad genetic characterization of HCU in Brazil, performing *CBS* analysis in HCU patients that are being followed at several centers nationwide.

## 2 METHODS

The present study was approved by the local research ethics committee (Hospital de Clínicas de Porto Alegre, Brazil). Collection procedures were conducted only after participants/legal guardians had agreed to take part in the investigation and provided written informed consent. To be included in the study, the patient should have been previously diagnosed as having HCU according to the following criteria: (1) presence of high levels of homocysteine in plasma; (2) presence of normal or high levels of methionine in plasma; AND (3) presence of clinical picture compatible with HCU. As public neonatal screening for HCU is not available in Brazil, all patients were late diagnosed (e.g., diagnosed after the starting of clinical manifestations).

### 2.1 Patients

The study sample comprised of 35 Brazilian HCU patients, from 30 different families. Families from 4/5 regions of Brazil were represented: south (*n* = 12), southeast (*n* = 11), northeast (*n* = 6), and midwest (*n* = 1) (Table 1). In addition, RNA samples were obtained from six patients. In 10 families, the genetic variant(s) found in the probands were also confirmed in at least one parent.

**Table 1:** *CBS* analysis—pathogenic mutations found in patients with classical homocystinuria (*n* = 35)

Patient	Sex	Origin (Brazilian region)	Allele 1		Allele 2		Cons.	Age at inclusion (years)	Age of onset (years)	B6 response found	B6 response expected^a^
			cDNA	Protein	cDNA	Protein					
1a	M	S	c.253G>A^c^	p.Gly85Arg	c.253G>A	p.Gly85Arg	Y	36	6	N	N
1b	F	S	c.253G>A^c^	p.Gly85Arg	c.253G>A	p.Gly85Arg	Y	27	NA	N	N
1c	F	S	c.253G>A^c^	p.Gly85Arg	c.253G>A	p.Gly85Arg	Y	31	7	N	N
2	M	S	c.833T>C	p.Ile278Thr	c.833T>C	p.Ile278Thr	Y	35	0.2	Y	Y
3	M	S	c.833T>C	p.Ile278Thr	c.833T>C	p.Ile278Thr	N	35	7	Y	Y
4	F	SE	c.833T>C^c^	p.Ile278Thr	c.833T>C^d^	p.Ile278Thr	Y	18	15	Y	Y
5	M	SE	c.833T>C	p.Ile278Thr	c.28delG	p.Val10 *fs*	N	26	1	Y	Y/N^b^
6	M	SE	c.833T>C	p.Ile278Thr	c.451G>A	p.Gly151Arg	N	23	4	Y	Y/N^b^
7	F	S	c.833T>C	p.Ile278Thr	**c.989_991delAGG**	**p.(Glu330del)**	N	28	20	N	Y/N^b^
8	M	NE	c.833T>C	p.Ile278Thr	c.828+1G>A	p.?	N	16	4	N	N^b^
9	F	S	c.828+1G>A^c^	p.?	c.1126G>A	p.Asp376Asn	N	23	5	N	N^b^
10	M	SE	c.828+1G>A^d^	p.?	**c.2T>C** c	**p.?**	N	13	1	N	N^b^
11	F	S	c.828+1G>A	p.?	c.691G>C	p.Ala231Leu	N	13	1	N	N^b^
12	M	NE	c.828+1G>A	p.?	c.828+1G>A	p.?	Y	8	1	N	N^b^
13	M	SE	c.572C>T	p.Thr191Met	c.572C>T	p.Thr191Met	Y	26	19	N	N
14	M	SE	c.572C>T	p.Thr191Met	c.572C>T	p.Thr191Met	Y	10	5	N	N
15	F	S	c.572C>T	p.Thr191Met	c.572C>T	p.Thr191Met	Y	18	5	N	N
16	M	SE	c.572C>T	p.Thr191Met	c.572C>T	p.Thr191Met	Y	19	4	N	N
17	M	S	c.572C>T	p.Thr191Met	**c.209+1delG** c	**p.?**	N	14	8	N	N^b^
18a	M	SE	c.969G>A^c^	p.Trp323Ter	c.969G>A^d^	p.Trp323Ter	Y	17	6	N	NA
18b	M	SE	c.969G>A^c^	p.Trp323Ter	c.969G>A^d^	p.Trp323Ter	Y	6	1	N	NA
19	M	NE	c.969G>A	p.Trp323Ter	c.969G>A	p.Trp323Ter	N	15	1	N	NA
20	M	NE	c.969G>A	p.Trp323Ter	c.969G>A	p.Trp323Ter	N	10	7	N	NA
21a	M	SE	c.451G>A	p.Gly151Arg	c.451G>A	p.Gly151Arg	Y	15	7.5	N	N
21b	F	SE	c.451G>A	p.Gly151Arg	c.451G>A	p.Gly151Arg	Y	14	NA	N	N
22	F	SE	c.451G>A	p.Gly151Arg	c.451G>A	p.Gly151Arg	Y	17	3	N	N
23	M	S	**c.284T>C** c	**p.Ile95Thr**	**c.284T>C**	**p.Ile95Thr**	Y	18	1	N	NA
24	M	S	c.1058C>T^c^	p.Thr353Met	c.146C>T	p.Pro49Leu	N	19	3	Y	Y^b^
25	F	S	c.1126G>A^c^	p.Asp376Asn	c.1126G>A^d^	p.Asp376Asn	Y	14	1.5	N	N
26	M	S	**c.444delG** c	**p.(Asn149** ***fs*** **)**	**c.444delG**	**p.(Asn149** ***fs*** **)**	Y	22	1.5	N	NA
27	M	NE	**c.329A>T**	**p.Glu110Val**	c.770C>T	p.Thr257Met	N	16	3	N	N^b^
28a	F	NE	**c.1223+5G>T**	**p.?**	**c.1223+5G>T**	**p.?**	Y	5	3	NA	NA
28b	F	NE	**c.1223+5G>T**	**p.?**	**c.1223+5G>T**	**p.?**	Y	7	3	NA	NA
29	F	CW	**c.864_868delGAG**	**p.(Glu289del)**	**c.864_868delGAG**	**p.(Glu289del)**	Y	17	6	N	NA
30	M	SE	c.209+1G>A	p.?	NI	NI	N	37	6	N	N^b^

Novel mutations are set in bold. Patients represented by the same number belong to the same family. M, male; F, female; S, south; SE, southeast; NE, northeast; CW, central‐west; Cons, consanguinity; Y, yes; N, no; B6, pyridoxine; NA, not available; NI, not identified.

a According to previously described cases in the literature (Kraus, 2017), all partially responsive patients were considered as nonresponsive.

b Estimated; no identical genotype reported previously.

c Mother heterozygous for mutation.

d Father heterozygous for mutation.

Patients were recruited through contact with physicians involved in care and/or research activities at medical genetics centers across the country. Overall, 13 medical centers participated in the study. Some patients have been followed in different medical centers in Brazil and even abroad throughout their lives. Thus, it may be possible that they might have been or will be studied and described elsewhere. To the best of our knowledge, however, only one patient in our sample (patient #30) might have been already described in the *CBS* mutation database (Kraus, 2017), although no clinical data is available there.

Pyridoxine responsiveness was the clinical parameter used to evaluate genotype–phenotype relationship. For the purposes of this study, patients were classified as responsive if they achieved target homocysteine levels (<100 μmol/L) on pyridoxine alone or pyridoxine + folic acid (regardless of the number of weeks since testing) (Morris et al., 2016). All other patients were classified as nonresponsive to pyridoxine. The genotypes found were compared with other family members and with previously reported cases.

### 2.2 *CBS* sequencing

Genomic DNA was extracted from whole blood using the commercially available Easy‐DNA™ gDNA Purification Kit (Invitrogen), following manufacturer instructions. Exons 1–14 and 16 and the exon/intron junctions of the *CBS* were amplified by conventional PCR, using primers and reaction conditions previously described elsewhere (Kruger, Wang, Jhee, Singh, & Elsas, 2003). The following primers were designed to amplify exon 15: forward, CCACAGGAAGAGTTGGGAGG; reverse, TGAGAGCCATTCTGAGGGGT. After amplification, fragments were purified and sequenced by the Sanger method. The sequence found was compared to the GenBank reference sequence (NG_008938.1). Any mutations identified were confirmed by repetition of amplification and sequencing reactions. Furthermore, parental DNA was used whenever available to confirm that mutations were in *trans* position.

Missense mutations not previously described in the literature were analyzed *in silico* in the PolyPhen2 (Polymorphism Phenotyping), MutPred, and SIFT (Sorting Intolerant From Tolerant) software. In addition, a group of 100 controls were tested for the novel c.2T>C (exon 1), c.284T>C (exon 2), and c.329A>T (exon 3) mutations and for the previously described c.828+1G>A mutation (intron 7). Testing for c.2T>C was performed by the restriction fragment length polymorphism (RFLP) method with the *NIaIII* restriction enzyme, whereas the other mutations were analyzed by sequencing of the mutation‐containing exon.

### 2.3 Homology modeling

Structural analyses were performed to investigate the structural and stability alteration of the novel coding *CBS* missense variants from the native CbS protein structure. The selected mutant models (c.284T>C; p.Ile95Thr and c.329 A>T; p.Glu110Val) were generated using SWISS‐MODEL and its automated server based on the target‐template alignment using ProMod3. The crystal structure of the CbS protein was retrieved from the Protein Data Bank (PDB ID 4COO, resolution at 2 Å) (McCorvie et al., 2014). Coordinates which are conserved between the target and the template are copied from the template to the model. Finally, the geometry of the resulting model is regularized by using a force field. In case loop modeling with ProMod3 fails, an alternative model is built with PROMOD‐II (Guex and Peitsch, 1997). The Swiss‐PDB viewer (version 4.1.0) was utilized for energy minimization of the modeled 3D structure.

### 2.4 qRT‐PCR

For qRT‐PCR analysis of gene expression, blood samples from six patients were collected into PAXgene tubes (Qiagen). RNA isolation was performed with the PAXgene Blood RNA kit (Qiagen) in accordance with manufacturer instructions. cDNA was then synthesized using the High‐Capacity cDNA Reverse Transcription Kit (Life Technologies). *CBS* mRNA levels were determined by qRT‐PCR using the commercially available *TaqMan Expression Assay* (Hs00163925_m1 (*CBS*); Hs02758991_g1 (*GAPDH*); and (Applied Biosystems) in a StepOne system (Applied Biosystems). *GAPDH* was used as the housekeeping gene. All reactions were performed under the conditions specified in the corresponding manufacturer instructions. Relative quantification of *CBS* RNA was normalized to the *GAPDH* gene using the 2^−∆∆CT^ method (Schmittgen & Livak, 2008).

### 2.5 Expression of mutations in *E. coli*

The novel mutations c.284T>C and c.329A>T were expressed in *E. coli* using a protocol adapted after Mendes et al., (2014), as described below.

For expression of wild‐type (WT) and mutant CβS, WT cDNA was first cloned in pOTB7 vector (Thermo Scientific, Lafayette, CO, USA), between restriction sites EcoRI and XhoI. The insert was then cleaved with NdeI and XhoI, purified (QIAquick gel extraction kit, Qiagen), and ligated into pET28b (Clontech Laboratories), at the same sites, with T4 DNA ligase (New England Biolabs). The pET28b carries an N‐terminal *6xHis‐tag*, followed by a thrombin cleavage site which enables later removal of this tag. The pET28b‐6xHis‐pepT‐hCBSWT expression construct was thus created and used as a template for site‐directed mutagenesis with the QuikChange II XL Site‐Directed Mutagenesis kit (Agilent Technologies), per manufacturer instructions.

WT and mutant CβS proteins were expressed in *E. coli* (BL21 DE3). Cells without the expression vector and cells harboring an empty expression vector were used as negative controls. Cells were cultured at 37°C in LB medium and selected with kanamycin. Protein expression was induced by adding isopropyl‐β‐D‐thiogalactoside (IPTG) and δ‐aminolevulinic acid (ALA) to the medium. After 16 hr at 22°C, bacteria were resuspended in lysis buffer and sonicated. The insoluble (pellet) and soluble (supernatant) fractions were separated by centrifugation.

The proteins thus generated were analyzed by SDS‐PAGE and Western blotting. Briefly, the protein content of the pellet fraction was quantitated by the Bradford method. Then, a 60‐μg aliquot of protein was analyzed by SDS‐PAGE. The same amount was used for Western blot analysis. This was performed using PVDF membranes, primary mouse anti‐CbS polyclonal antibody (Abnova, A75‐A01), and secondary polyclonal rabbit anti‐His tag antibody (PAB0862, Abnova). The enzyme activity of CβS was determined by LC‐MS/MS in the soluble fraction of the lysate, using the protocol described by Smith et al., (2012). All experiments were performed in triplicate, with the arithmetic mean of the resulting measurements considered for analysis.

### 2.6 Statistical analysis

Statistical analysis was performed using SPSS for Windows, Version 18.0 (Chicago: SPSS Inc). Asymmetrically distributed variables were expressed as median (range), and normally distributed variables as mean and standard deviation. The Mann–Whitney *U* test (continuous variables) or chi‐square test (categorical variables) was used to assess between‐group differences. *p*‐values <.05 were considered significant. To calculate allele frequencies, only unrelated patients were considered (*n* = 30), and when consanguinity was reported, only one allele per patient was taken into account.

## 3 RESULTS

The summary of the clinical and genetic data of the sample is shown in Table 1.

Of the 35 patients included, 22 (63%) were male. Parental consanguinity was reported in 16 families (53%). Median age at inclusion was 19 years (range 5–37 years). Regarding pyridoxine responsiveness, 28 patients (85%) were classified as nonresponsive and five (15%) as responsive, with three of them homozygous for the p.Ile278Thr mutation (patients 4, 5, and 6). In two patients, pyridoxine responsiveness was not reported/determined. The median age of symptom onset was 4 years (Table 1).

### 3.1 Genotype

**Figure 1:**
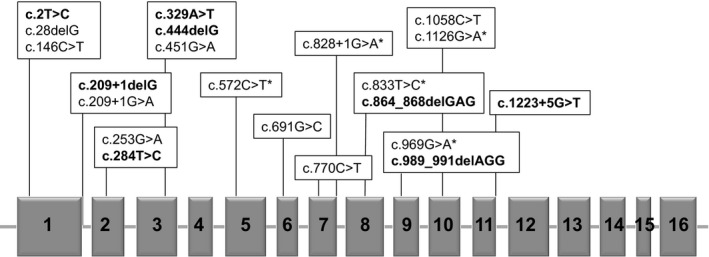
*CBS* map showing the location of the mutations found. Exons are represented by solid gray boxes. Novel mutations are shown in bold. An asterisk (*) indicates mutations found in more than one unrelated patient. Adapted from Kraus, 2017 (Kraus, 2017)

Twenty‐one different mutations were detected, with six recurrent (Figure 1). Regarding the type, most mutations were missense (*n* = 11, nine transitions and two transversions), followed by splicing site (*n* = 4), small deletions (*n* = 2), frameshift (*n* = 2) and nonsense (*n* = 1).

Exons 8, 9, 7, 5, and 3 had the higher number of nonrelated alleles mutated (*n* = 9, 7, 6, 5, and 5, respectively). Altogether, the most prevalent mutations were: p.Ile278Thr (allele frequency 18.2%; found in south, southeast, and northeast regions), p.Trp323Ter (allele frequency 11.3%; found in southeast and northeast), p.Thr191Met (allele frequency 11.3%; found in south and southeast), and c.828+1G>A (allele frequency 11.3%; found in south, southeast, and northeast). In only one allele no mutation could be identified (patient 30; Table 1). Eight novel mutations were detected: c.2T>C (exon 1), c.209+1delG (intron 1), c.284T>C (p.Ile95Thr, exon 2), c.329A>T (p.Glu110Val, exon 3), c.444delG (exon 3), c.864_868delGAG (exon 8), c.989_991delAGG (exon 9), and c.1223+5G>T (intron 11). No mutant alleles were detected in the 100 controls tested.

### 3.2 Genotype versus phenotype relationship

When available in the literature, the expected phenotype according to the genotype (responsive or nonresponsive to pyridoxine) matched in all cases the observed phenotypes (Table 1).

All patients from the same family (1, 18, 21, and 28) presented the same phenotype regarding pyridoxine responsiveness. All homozygous for the p.Ile278Thr mutation (*n* = 3) were pyridoxine responsive. In the compound heterozygous (*n* = 3), association with another missense mutation (p.Gly151Arg) resulted in pyridoxine responsiveness in patient #6, while patients #7 and #8, carrying the mutations c.989_991delAGG and c.828+1G>A, were nonresponsive.

Most patients (4/5) carrying the p.Thr191Met mutation were homozygous, and all five were nonresponsive to pyridoxine. Patient #17 is heterozygous for the p.Thr191Met and the novel c.209+1delG mutation.

Patients carrying the c.828+1G>A were also nonresponsive to pyridoxine. Most of them (4/5) were compound heterozygous, and 3/4 carried a missense mutation in the second allele. Patients carrying the p.Trp323Ter mutation (*n* = 4) were all homozygous and nonresponsive to pyridoxine.

### 3.3 In silico analysis

The p.Ile95Thr and p.Glu110Val mutations were analyzed in the PolyPhen‐2 and SIFT software for prediction of functional effects, and both were predicted to be pathogenic (PolyPhen‐2: scores of 0.999 and 1.000, respectively; SIFT: score of 0 for both mutations). The MutPred software suite, which estimates potential changes in mutant proteins, interpreted both mutations as probably damaging, with scores of 0.827 for p.Ile95Thr and 0.892 for p.Glu110Val.

In the crystal structure of the human CbS enzyme, Glu110 and Ile95 residues are located in the catalytic domain and have a predicted severe effect. Ile95 is a conserved residue located in the H3 of the catalytic domain near the dimers interface (Figs S1 and S2); its main interactions are with Phe99 and Lys98 residues located in the same helix and Glu342 located in the H14. The alignment of the structure of the mutant model I95T with the CbS protein structure (PDB:4COO) showed no alteration of the original interactions of Ile95 in the monomers (Fig. S1). Glu110 is located in the H4 near to a highly conserved position surrounding the cofactor pyridoxal 5‐phosphate (PLP). Glu110 form hydrogen bonds with the following residues: Thre87 located next to the strand A, Asn113 located in a beta turn, and Arg121 located in the H5. Salt bridge is observed with Lys108 and Arg121 residues. The central part of the dimer interface is formed by the residues Phe111 and Phe112 close to the twofold dimer axis; thus, Phe112 of monomer A interacts with Phe112 of monomer B and vice versa (Meier et al., 2001). The side chain of the Thr87 is part of the dimer interface. Alignment of the mutant model Glu110V with 4COO showed a loss of interaction with the Thr87 (Figs S1 and S2).

### 3.4 Expression studies

**Figure 2:**
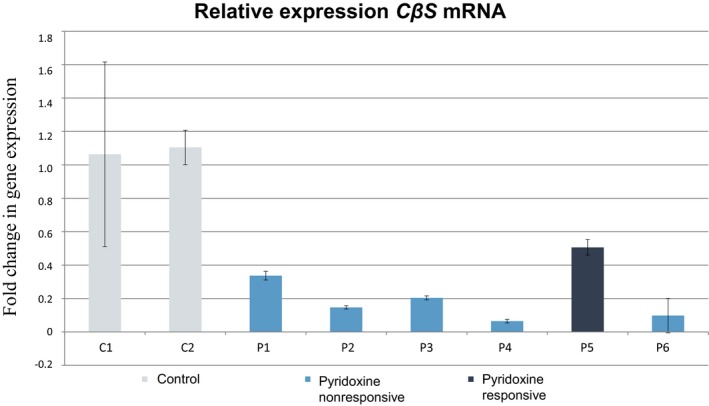
*CBS* mRNA expression as determined by qRT‐PCR. Genotypes: P1 (c.209+1delG; c.572C>T); P2 (c.2T>C; c.828+1G>A); P3 (c.828+1G>A; c.1126G>A); P4 (c.833T>C; c.989_991delAGG); P5 (c.1058C>T; c.146C>T); and P6 (c.444delG; c.444delG). Fold change in gene expression is calculated through the 2^−∆∆^ ^*C*^_T_ method, where ∆*C*_T_ = (*C*_T__,Time x_ *‐* *C*_T__,Time 0_) (Schmittgen & Livak, 2008)

Enzyme activity in the *E. coli‐*expressed mutant proteins in relation to the WT control was 1.5% (24.7 nmol/h/mg protein) for c.329A>T (p.Glu110Val) and 17.5% (432.6 nmol/h/mg protein) for c.284T>C (p.Ile95Thr). Relative *CBS* mRNA levels measured by qRT‐PCR in six patients are described in Figure 2. Reduced expression was observed in all, with the highest expression level detected in the sole pyridoxine‐responsive patient (P24).

## 4 DISCUSSION

The present report provides the largest genetic analysis of Brazilian HCU patients reported so far. The *CBS* gene was sequenced for 35 patients (30 unrelated) followed in 13 medical genetics centers across Brazil. Age of symptom onset varied considerably within the sample. However, more than half of the patients presented symptoms at an early age (<5 years old), suggesting more severe forms of HCU in these patients. All patients included in the study have had delayed diagnosis, based mainly on clinical suspicion. This is partially explained by the fact that, in Brazil, HCU is not included in the National Neonatal Screening Program.

A high proportion of pyridoxine‐nonresponsive HCU was observed in the study population. Nonresponsiveness to pyridoxine is associated with a more severe phenotype and challenging management (Mudd et al., 1985, 2001; Wilcken, 2006). In our study, this proportion exceeded rates described worldwide (approximately 50%) (Mudd et al., 2001). Although the proportion of nonresponsive patients is also high in some countries, such as Qatar and Ireland, these cases are associated with specific genotypes that are highly prevalent in the corresponding countries (El‐Said et al., 2006; Gallagher et al., 1995). No such association was observed in our study. In fact, the most prevalent mutation in our sample (p.Ile278Thr) is a pyridoxine‐responsive mutation. These findings may suggest that milder, pyridoxine‐responsive forms of HCU remain largely underdiagnosed in Brasil.

Great variability in genotypes was observed in the present study. The Brazilian population is characterized by extraordinary genetic diversity as a result of centuries of admixture among Amerindians, European colonizers, and African slaves (IBGE, 2007). European ancestry has the higher contribution to the genetic background of Brazilians (0.62), followed by African (0.21) and Amerindian (0.17) (Moura, Coelho, Balbino Vde, Crovella, & Brandao, 2015). However, major regional differences exist: European contributions are even more dominant in the south of the country, whereas the northeast and north regions have the highest proportions of African and indigenous ancestry, respectively (Moura et al., 2015; Ruiz‐Linares et al., 2014). This might contribute to the wide range of genotypes found. In our study, all frequent mutations found were detected in at least two different regions of Brazil, which does not support the hypothesis that they could have arisen through a founder effect.

The most prevalent mutation in our study was the p.Ile278Thr (c.833T>C). This is also the most prevalent mutation worldwide, accounting for 16% of all HCU alleles described (Kraus, 2017). It is particularly frequent in central and northern Europe (Kluijtmans et al., 1999; Sebastio et al., 1995; Shih et al., 1995; Sokolova et al., 2001; Sperandeo et al., 1995). In the Brazilian study conducted by Porto et al., this mutation was detected in 6 of 28 alleles (frequency in unrelated alleles, 13.6%) (Porto et al., 2005). Since patients carrying this mutation can exhibit very mild or isolated symptoms later in life, it is possible that the prevalence of this mutation in Brazil is even higher, and that many HCU patients may remain undiagnosed. In the present study, most patients harboring this mutation were pyridoxine responsive (4/6), with the two patients classified as nonresponsive being compound heterozygous carrying more severe mutations in the second allele. Although less frequent, several other cases of pyridoxine‐nonresponsive patients carrying the p.Ile278Thr mutation are described (Kluijtmans et al., 1999; Kraus, 2017; Porto et al., 2005).

The Iberian mutation p.Thr191Met (c.572C>T) was found in 11.3% of unrelated alleles, again all in patients from the south and southeast regions. This allele frequency was similar to that reported by Porto et al. (13.64%) (Porto et al., 2005), but lower than those reported in other Latin American countries (75% in Colombia, 25% in Venezuela, and 20% in Argentina) and in the Iberian Peninsula (52% in Spain and 33% in Portugal) (Bermudez et al., 2006; Cozar et al., 2011; Urreizti et al., 2003). These findings appear to reflect the greater genetic heterogeneity of Brazil as compared with other Latin American countries and, possibly, the more limited contribution of Spanish immigration to Brazil (IBGE, 2007; Kehdy et al., 2015; Pena et al., 2011; Resque et al., 2016). Wide phenotypic variability has been observed for this mutation, with mild to severe phenotypes and pyridoxine responsiveness ranging from partial to absent (Kraus, 2017; Urreizti et al., 2006).

The c.828+1G>A mutation, detected in 11.3% of the alleles in our studies, had previously been described in only one individual, a heterozygous Czech patient [c.1146‐2A>C; c.828+1G>A] (Janosik et al., 2001). Their patient was described as pyridoxine nonresponsive and had null CβS activity in fibroblasts. Furthermore, there was no mRNA expression of the allele containing the mutation. According to the authors, this suggests a nonsense‐mediated mRNA decay mechanism, as a premature termination codon at exon 8 has been predicted for this mutation (Janosik et al., 2001). In our study, all patients with this mutation were classified as nonresponsive. One patient heterozygous for (c.828+1G>A; c.1126G>A) underwent qRT‐PCR analysis, which showed a ~80% reduction in mRNA expression. Our clinical data support the severity of this mutation. In our sample, all patients carrying the c.828+1G>A presented early symptom onset (≤5 years) and were all nonresponsive to pyridoxine, even the compound heterozygous. The high prevalence of this mutation in Brazilian patients does not appear to be related to genetic drift effects, as the affected patients came from three geographically distant regions of the country. Furthermore, this mutation was not detected in any of the 100 healthy controls, and thus appears to be rare in the overall population.

The p.Trp323Ter (c.969G>A) mutation was also detected with an allele frequency of 11.3%. This mutation is highly prevalent in Saudi Arabia (10 of 13 families assessed) and is associated with severe phenotypes (Zaidi et al., 2012). In our study, all patients with this mutation were homozygous and pyridoxine nonresponsive. These families were from the northeast and southeast regions of Brazil. As there was no determination of ancestry in our study, we could not infer whether the presence of this mutation in Brazilian patients might be associated with migratory events.

Eight novel mutations were detected in this study. Several tests corroborated the pathogenicity of the analyzed missense mutations. In silico analyses using three different software programmes predicted a damaging functional effect for the mutations p.Glu110Val and p.Ile95Thr. Furthermore, no allele containing these mutations was detected in 100 controls. *E. coli* expression assays demonstrated reduced enzyme activity consistent with pathogenicity for HCU of both mutations (<20% activity relative to controls) (Arruda et al., 1998; Picker & Levy, 2014). Enzyme activity in *E. coli* also correlated well with clinical phenotype (pyridoxine responsiveness and disease severity).

Homology modeling of the p.Ile95Thr mutation showed no alteration of the original interactions of Ile95 in the monomers. However, the loss of hydrophobicity due to the substitution of Ile to Thr may affect the hydrophobic character of the dimer interface. This could explain the lack of residual activity of the mutant in the protein extract. Analysis of protein oligomerization and CbS enzyme activity of the purified mutant protein could provide more information to confirm the structural predictions.

For the p.Glu110Val mutation, structural analyses showed that the substitution of Glu to Val may disturb the ionic interactions due to the charge difference of the Glu (negative) and Val (neutral). Furthermore, the size and the hydrophobicity difference may affect the hydrogen bond formation with the surrounded residues remaining just the interaction with Asn113 and Lys1083.

The c.2T>C mutation alters the translation start site codon, being the first of its kind ever described in *CBS* (Kraus, 2017). This mutation was not detected in the 100 controls we tested and not found in the 1000 genomes database (http://www.1000genomes.org/). Start‐site missense mutations are relatively common in hereditary diseases; in fact, point mutations at this position are more likely to be damaging than other missense mutations (Wolf et al., 2011). In *CBS*, the next ATG codon is located in exon 2, far from the first methionine codon. However, exon 1 carries a leucine at position 28 with a flanking sequence that approximates the “Kozak consensus sequence,” a specific flanking sequence that maximizes translational efficiency (Kozak, 2002; Wolf et al., 2011). This suggests that this CTG codon could be used as an alternative initiation codon in *CBS*.

Finally, qRT‐PCR analysis of samples from four patients heterozygous for the novel c.2T>C, c.209+1delG, c.989_991delAGG, and c.444delG mutations revealed major reductions in mRNA levels relative to controls, which suggests reduced gene expression in the presence of these variants. This reduction is greater in patients with splice‐site mutations, which disrupt reading, as premature termination codons are encoded in the mutant sequence; therefore, mRNA decay pathways eventually degrade the product of expression. Our findings were also consistent with the most severe phenotypes being found in patients with greater reductions in mRNA levels.

In general, the genotypes were consistently associated with pyridoxine responsiveness (presence or absence) within families and also agreed with previous findings worldwide (Janosik et al., 2001; Kraus, 2017; Zaidi et al., 2012). However, it is important to highlight that no clear genotype–phenotype correlation is established for most *CBS* mutations. The lack of clinical data in many studies, varied pyridoxine responsiveness protocols, and the high number of private/rare mutations in *CBS* are some of the factors that limit this analysis.

In our study, 72% of the mutated alleles were condensed in just five exons. Considering this finding, we propose a protocol for *CBS* sequencing that would be cost‐ and time‐saving for the molecular investigation of new Brazilian HCU patients. We suggest that exons 8, 9, 7, 5, and 3 are sequenced first, since they carried the great majority of mutated alleles found in our sample. Second, exons 1, 2, 4, 10, 11, 12, and 16 should be tested. In our study, 10 different mutations were found there, and a great number of mutations reported worldwide are also located in these exons. We suggest that exons 6, 13, 14, and 16 are sequenced last, since mutations in these regions are rare. No disease‐causing mutation has been reported in exon 15 (Kraus, 2017).

In conclusion, this study provides the most wide‐ranging genetic characterization of HCU in Brazil to date. Most patients studied here were nonresponsive to pyridoxine and presented clinically early in life, suggesting more severe forms of HCU in our sample. A great variability in genotypes was observed; this might reflect the intense admixture and the diverse genetic background of the Brazilian population. The four most prevalent mutations together accounted for over 50% of mutated alleles. A consistent genotype–phenotype association was observed within families and for common mutation. However, for many rare and novel mutations described here, additional studies should be carried to evaluate the effect of these variants on human CβS deficiency. These findings should contribute to the development of protocols for diagnosis and molecular screening of HCU in Brazil.

## CONFLICT OF INTEREST

The authors declare no conflict of interest.
