# Supplementary figures and images for: CBS mutations are good predictors for B6‐responsiveness: A study based on the analysis of 35 Brazilian Classical Homocystinuria patients
Source: Mol Genet Genomic Med. 2018 Jan 20;6(2):160–70. doi: 10.1002/mgg3.342 (PMC5902399; doi:10.1002/mgg3.342)

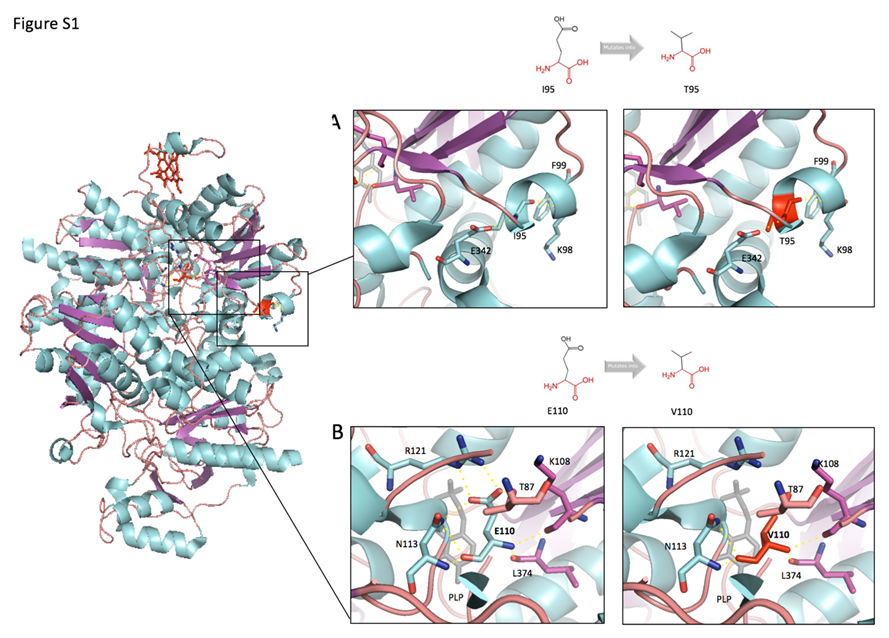

Supplement: Supplementary file 1 [file MGG3-6-160-s001.tif]
