# Supplementary material for: CBS mutations are good predictors for B6‐responsiveness: A study based on the analysis of 35 Brazilian Classical Homocystinuria patients
Source: Mol Genet Genomic Med. 2018 Jan 20;6(2):160–70. doi: 10.1002/mgg3.342 (PMC5902399; doi:10.1002/mgg3.342)
Supplement: Supplementary file 2 [file MGG3-6-160-s002.pdf]

S2 A) Figure

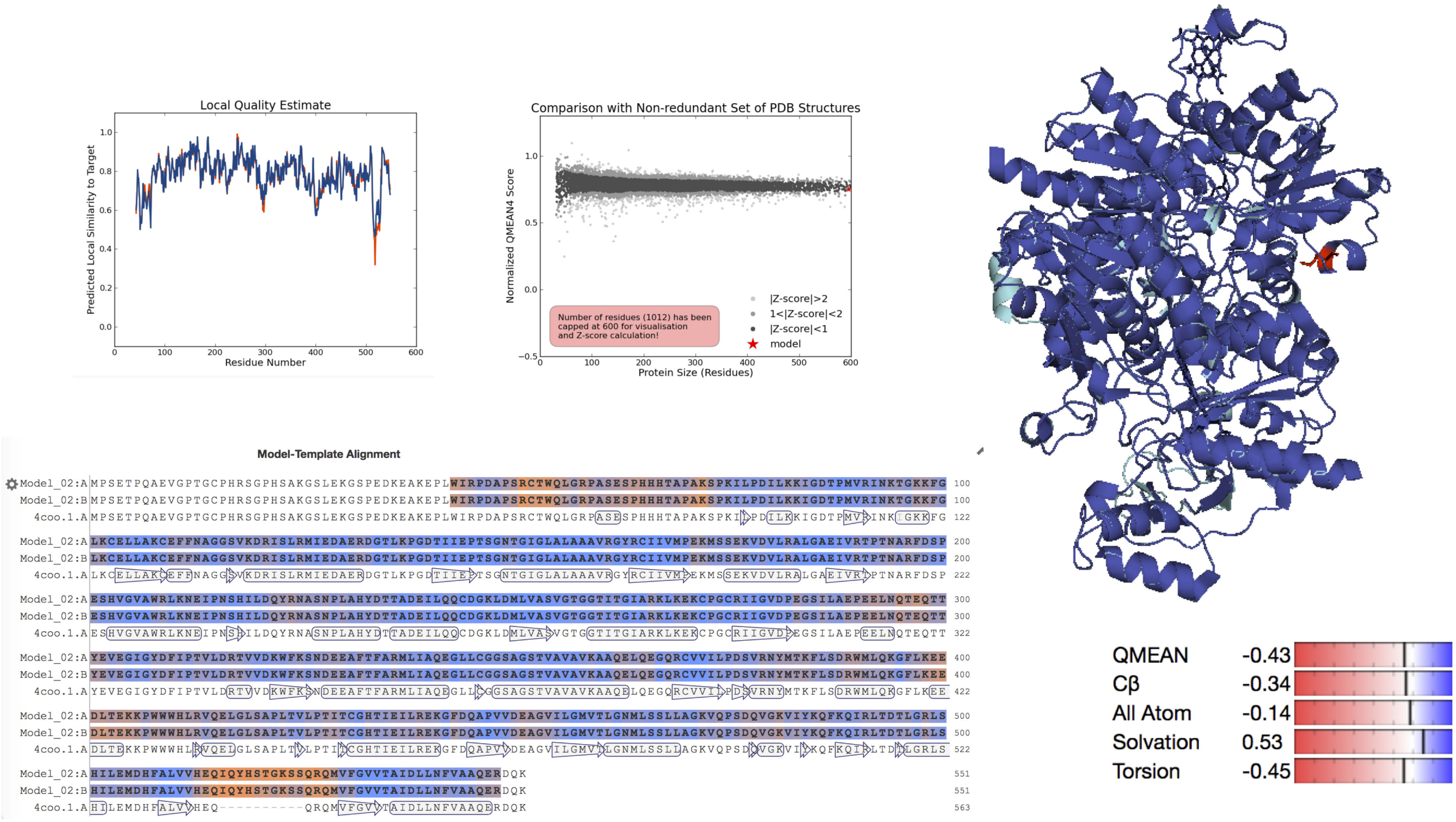

Template	Seq Identity	Oligo-state	Found by	Method	Resolution	Seq Similarity	Range	Coverage	Description
4coo.1.A	99.82	homo-dimer	BLAST	X-ray	2.00Å	0.61	43 - 548	0.98	CYSTATHIONINE BETA-SYNTHASE

S2 B) Figure

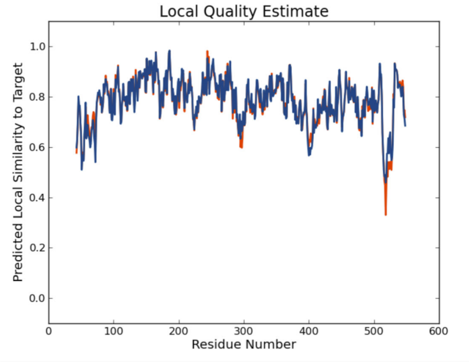

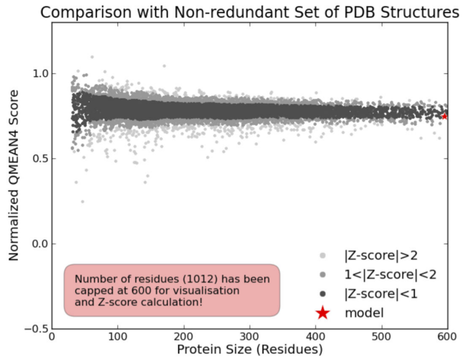

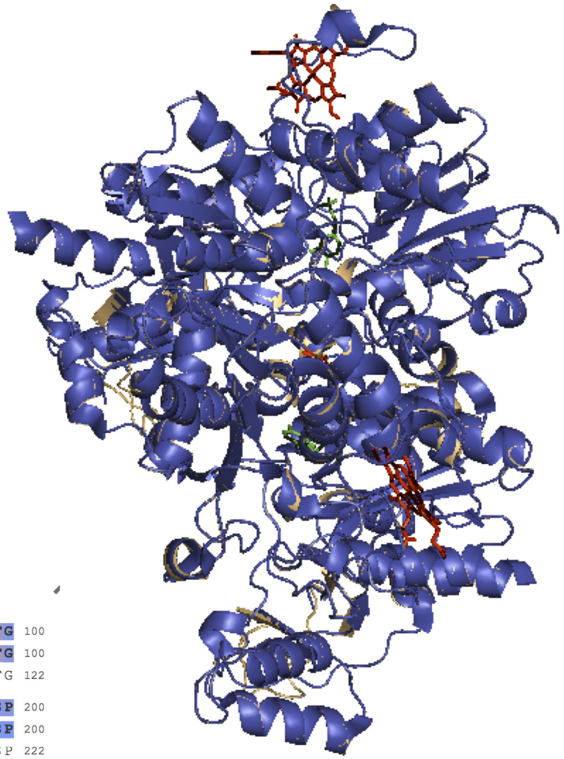

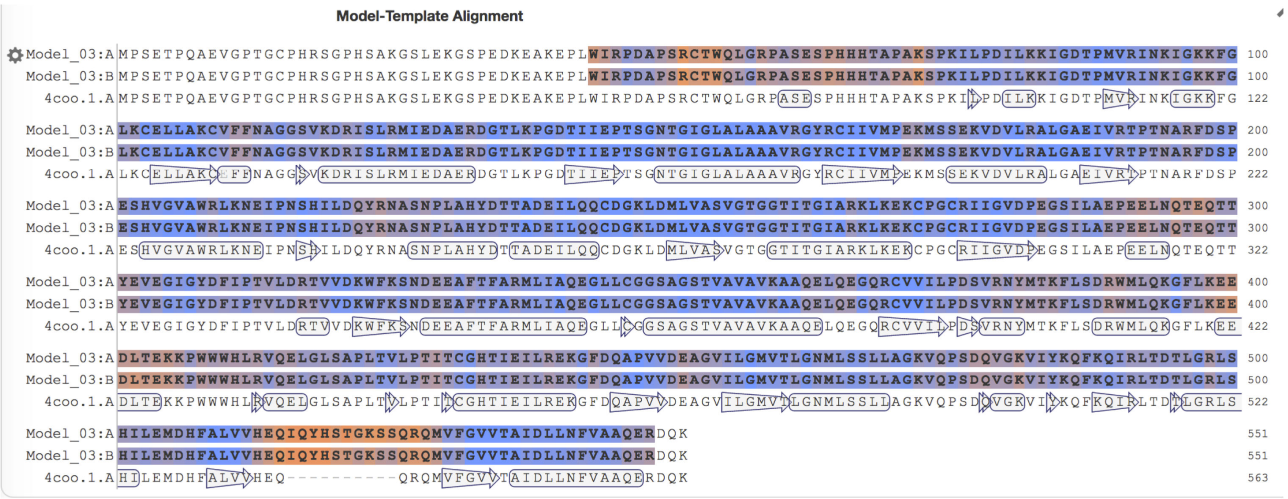

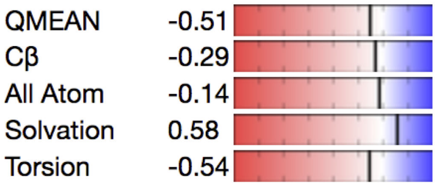

Template	Seq Identity	Oligo-state	Found by	Method	Resolution	Seq Similarity	Range	Coverage	Description
4coo.1.A	99.82	homo-dimer	BLAST	X-ray	2.00Å	0.61	43 - 548	0.98	CYSTATHIONINE BETA-SYNTHASE
